# Is good muscle function a protective factor for early signs of knee osteoarthritis after anterior cruciate ligament reconstruction? The SHIELD cohort study protocol

**DOI:** 10.1016/j.ocarto.2020.100102

**Published:** 2020-09-24

**Authors:** Anna Cronström, May Arna Risberg, Martin Englund, Carl Johan Tiderius, Patrik Önnerfjord, André Struglics, Jonas Svensson, Pernilla Peterson, Sven Månsson, Eva Ageberg

**Affiliations:** a Department of Health Sciences, Lund University, Lund, Sweden; b Department of Community Medicine and Rehabilitation, Umeå University, Umeå, Sweden; c Division of Orthopedic Surgery, Oslo University Hospital, Oslo, Norway; d Department of Sports Medicine, Norwegian School Sport Sciences, Oslo, Norway; e Lund University, Department of Clinical Sciences Lund, Orthopaedics, Clinical Epidemiology Unit, Lund, Sweden; f Lund University, Department of Clinical Sciences Lund, Orthopaedics, Lund, Sweden; g Lund University, Department of Clinical Sciences Lund, Rheumatology and Molecular Skeletal Biology, Lund, Sweden; h Medical Radiation Physics, Department of Translational Medicine, Lund University, Skåne University Hospital, Malmö, Sweden; i Imaging and Physiology, Skåne University Hospital, Lund, Sweden

**Keywords:** Osteoarthritis, ACL injury, Muscle function, Prevention

## Abstract

**Introduction:**

Knee injury history and increased joint load, respectively, are major risk factors for the development of knee osteoarthritis (OA). Lower extremity muscle function, such as knee muscle strength, influence joint load and may be important for the onset of knee OA. However, the role of muscle function as a possible modifiable protective mechanism for the development of OA after anterior cruciate ligament reconstruction (ACLR) is not clear.

**Methods and analysis:**

In this prospective cohort study, 100 patients (50% women, 18–35 years) with ACLR will be recruited from Skåne University Hospital, Sweden and Oslo University Hospital, Norway. They will be assessed with a comprehensive test battery of muscle function including muscle strength, muscle activation, hop performance, and postural orientation as well as patient-reported outcomes, one year (baseline) and three years (follow-up) after ACLR. Primary predictor will be knee extension strength, primary outcome will be patient-reported knee pain (Knee injury and Osteoarthritis Outcome Score, subscale pain) and secondary outcomes include compositional MRI (T2 mapping) and turnover of cartilage and bone biomarkers. Separate linear regression model will be used to elucidate the influence of each baseline muscle function variable on the outcomes at follow-up, adjusted for baseline values. Twenty non-injured individuals will also be assessed with MRI. This study is approved by The Regional Ethical Review Board in Lund (Sweden) and Oslo (Norway).

**Discussion:**

This study may have important clinical implications for using muscle function to screen for risk of early-onset knee OA and for optimizing exercise therapy after knee injury.

## 1 Introduction

An anterior cruciate ligament (ACL) injury is commonly associated with long-term functional limitations [[1], [2], [3], [4]]. In addition, it is well established that a history of knee injury is a major risk factor for the development of knee osteoarthritis (OA). A recent systematic review and meta-analysis found that a previous ACL injury was associated with a four to six-fold increased risk of developing knee OA two to 22 years post knee injury [5].

OA develops slowly. Follow-up of approximately 5–10 years is required to detect radiographic features indicative of OA such as joint space narrowing and osteophytes, i.e., features found relatively late in the disease process [6], indicating that radiography is not useful for early detection of OA [7,8]. Early detection of the disease is important as this may permit early intervention, such as load management by appropriate exercise programs, before the biomechanical derangement becomes too severe. Patient-reported pain may be used as a marker of future significant knee pain and early symptomatic knee OA in patients with ACL injury [9,10] and has also been reported to be related to features of tibiofemoral OA, such as joint space narrowing and cartilage thickness [9,11,12]. Magnetic resonance imaging (MRI) techniques, such as T2 relaxation time (a measure of water content and biochemical composition) is another promising technique that seems sensitive for detecting cartilage degeneration early (two – three years) in the disease process [13,14]. T2 is reported to be higher in the involved knee 6–24 months after injury compared to both healthy controls [15] and to the contra-lateral knee [16]. Furthermore, increased knee adduction moment [17], worse self-reported activity of daily living [16] and lower knee muscle strength [18] seem to be associated with higher T2. Thus, T2 may be a clinically important measure of early knee OA after knee injury. Molecular biomarkers may also be used to identify early OA in these patients and several biomarker candidates are under validation [[19], [20], [21]].

Knee joint load is suggested to be a contributing factor for knee OA development and progression [[22], [23], [24], [25]]. Sensorimotor function, such as muscle strength and muscle activation patterns, factors that are modifiable by training, influences the magnitude of joint load and may, thus, play an important role for the onset of knee OA [26]. Knee muscle weakness has been reported to be associated with an increased risk of developing radiographic and symptomatic knee OA in individuals without previous knee joint injury [27], whereas the evidence for such association in individuals with a knee injury is inconclusive [27,28]. A recent systematic review reported conflicting results of other measures of muscle function than knee muscle strength, such as hop performance, as contributing factors for radiographic or symptomatic OA development after knee injury [28]. Furthermore, low patient-reported function 2 years after ACLR has also shown to be associated with symptomatic and radiographic knee OA ten years post injury [28]. To our knowledge, there are no studies on the role of muscle function as a possible modifiable protective mechanism for the development of early-stage OA after knee injury, using a comprehensive test battery of muscle function as well as different measures of early knee OA. Such knowledge would help in designing more effective training interventions in the treatment of knee injury and subsequent early-onset OA. Thus, the aim of this prospective cohort study including young people with anterior cruciate ligament reconstruction (ACLR) is to study the association between different measures of muscle function and early future knee OA assessed as patient-reported pain, features indicative of early radiographic OA measured with MRI, and early detection of OA in biomarker profiles, respectively. In addition, we will investigate the prevalence of early knee OA in individuals 1 and 3 years post ACLR, respectively, compared to non-injured controls.

## 2 Methods and analysis

This is a prospective cohort study adhering to the STROBE guidelines (http://www.strobe-statement.org). The study is registered on ClinicalTrials.gov (NCT03473873).

### 2.1 Participant and setting

Patients will be consecutively recruited from the Department of Orthopedics, Skåne University Hospital, Sweden (main site) and the Department of Orthopedics, Oslo University Hospital, Oslo, Norway. The majority of patients (approx. 70%) will be included in Sweden. The study is approved by The Regional Ethical Review Board in Lund, Sweden (Dnr 2016/319) and Oslo, Norway (REK sør-øst D: 2016/1128).

*Inclusion criteria*: i) one year (between 10 and 16 month) after ACLR, with or without associated injuries to other structures of the knee (e.g., collateral ligament(s), meniscal injury), ii) age 18–35 years. *Exclusion criteria*: i) previous serious injury or surgery to either knee (e.g., ACL tear, meniscal tear), ii) diseases or disorders overriding the knee condition (e.g., neurological disease), iii) contra-indicators for MRI, iv) not understanding the languages of interest (any Scandinavian language or English). Possible confounders to record at the assessments include: i) additional surgery to the index knee between the time point of ACLR and inclusion and/or during follow-up, ii) serious injury to the index knee (e.g., giving-way episode(s)) resulting in pain and/or swelling and requiring inpatient or outpatient health care between the time point of ACLR and inclusion and/or during follow-up. Demographics (e.g., age sex, BMI, heredity) and other patient-reported outcomes will be collected at baseline and at follow-up (See Table 1 for full details). As a sample of convenience, 20 age- and sex-matched non-knee injured individuals will be recruited among students in Lund, Sweden. With the exception that these individuals should be free from ACL injuries to both knees, the inclusion/exclusion criteria will be the same for this cohort as for the patient cohort.

**Table 1:** Demographics and patient-reported outcomes to be collected at baseline and at follow-up.

	Baseline		Follow-up	
	ACLR patients	Controls	ACLR patients	Controls
**Demographics**				
Sex	X	X		
Age (years)	X	X	X	X
Height (cm)	X	X		
Body mass (kg)	X	X	X	X
Primary sport	X	X		
years in primary sport	X	X		
Injured knee (left/right)	X			
Date of injury	X			
Date of reconstruction	X			
Graft type	X			
Injury situation	X			
Contact/non-contact injury	X			
Associated injuries (e.g., collateral ligament(s), meniscal injury) to the index knee	X			
Additional surgery to the index knee between the time point of ACLR and inclusion and/or during follow-up	X		X	X
Serious injury to the index knee (e.g., giving-way episode(s)) resulting in pain and/or swelling and requiring inpatient or outpatient health care between the time point of ACLR and inclusion and/or during follow-up	X		X	X
Current pain (numeric rating scale, 0 (none) to 10 (worst))	X		X	
Tegner activity scale before injury	X			
Tegner activity scale, current	X	X	X	X
OA heredity	X	X		
ACL injury heredity	X	X		
**Questionnaires**				
Knee injury and Osteoarthritis Outcome Score (all subscales)	X		X	
Short-Form 36^a^	X		X	
ACL return to sport after injury scale^a^	X		X	
ACL quality of life^a^	X		X	
Global knee function: numeric rating scale (NRS), 1 (worst) to 10 (best)	X	X	X	X
Return to sport (yes/no, level)	X		X	
Perceived Stress Scale - 10	X	X	X	X

ACLR = anterior cruciate ligament reconstruction, OA = osteoarthritis, age will be derived from the Swedish personal identity number, body mass and height will be assessed with anthropometric measures, associated injuries, additional surgeries, and additional injuries will be derived from medical records. All other measures are self-reported.

a Data collection in Lund only.

### 2.2 Assessments

All predictors and outcomes will be assessed at 1 year (baseline) and at 3 years (follow-up) after ACLR. *Primary predictor* is knee extension muscle strength at baseline. *Secondary predictors* are knee flexion, hip, and trunk muscle strength, hop performance, postural orientation and patient-reported outcomes (except patient-reported pain) at baseline. *Exploratory predictor* is muscle activation pattern at baseline. *Main outcome* is the Knee injury and Osteoarthritis Outcomes Score (KOOS), subscale pain at follow-up. *Secondary outcome* is T2 (MRI) and biomarker profiles at follow-up (full description of predictors and outcomes are outlined below and in Table 1, Table 2). One physical therapist researcher will perform all functional assessments in Lund and another physical therapist researcher will perform all functional assessments in Oslo. The researchers will give standardized verbal instructions and an inter-rater reliability testing has been performed, showing moderate to excellent inter-rater reliability for all functional assessments (ICC_2*K*_ 0.66–1.0) (See Appendix A for detailed description of the reliability assessment).

**Table 2:** Predictors and outcomes at baseline and follow up.

Predictors at baseline and follow-up for ACLR patients	Data collection instrument
**Primary predictor**	
Knee extensor muscle strength	Isokinetic strength (Nm/kg) (Biodex)
**Secondary predictors**	
Performance-based measures	Hop performance (side-hop (n), SLHD (cm)), Postural orientation errors (movement quality scoring)
Muscle strength	Isokinetic knee flexion strength (Biodex), isometric hip and trunk muscle strength (handheld dynamometer)
Patient-reported function	Questionnaires: KOOS subscales (except pain), SF-36, Tegner activity scale, ACL-RSI, ACL-QoL, Global knee function (NRS 1-10), PSS-10
**Exploratory predictor**	
Muscle activation pattern^a^	Muscle activation pattern of the hip, trunk and knee during the SLHD (electromyography)
**Outcomes at baseline and follow-up for ACLR patients and controls**	
**Primary outcome**	
Patient-reported outcome	Questionnaire: KOOS subscale pain
**Secondary outcomes**	
Quantitative assessment of cartilage morphology	T2 mapping (MRI)
Molecular biomarkers of cartilage and bone turnover assessed in serum	Blood samples of venous blood to assess specific biomarkers: COMP and ARGS-aggrecan, NTX-I, TRAP5b and cytokines

SLHD = single-leg hop for distance, KOOS = knee osteoarthritis outcome score, ACL-RSI = ACL Return to Sport after Injury Scale, ACL-QoL = ACL Quality of Life, SACQ = Self-administered Comorbidity Questionnaire, PSS-10 = Perceived Stress Scale, MRI = magnetic resonance imaging, ARGS-aggrecan = ARGS neoepitope of aggrecan, COMP = Cartilage oligomeric matrix protein, NTX-I = N-terminal type I collagen cross-linked telopeptide, TRAP5b = tartrate-resistant acid phosphatase 5b.

a Data collection in Lund only.

The participants are told to wear shorts and t-shirt. Strength tests will be performed without shoes whereas the postural orientation tasks and hop tests will be performed with training shoes. In an effort to minimize bias related to learning effects of the injured leg, the right leg will be tested first (irrespective of whether the right or left leg is injured). This will be applied for all assessments except for isokinetic knee strength for which the non-injured leg will be tested first, according to the standard procedure for Biodex assessments.

### 2.3 Predictors

#### 2.3.1 Knee strength

Isokinetic concentric muscle strength tests during knee extension and flexion will be measured at 60°/sec with a dynamometer (Biodex Medical Systems, Shirley, New York). Four trial repetitions will be performed with submaximal effort, followed by a 1-min rest. Five test repetitions will then be performed and recorded. Peak torque (Nm) and normalized peak torque (Nm/kg) for knee extension and flexion will be recorded [29].

#### 2.3.2 Hip and trunk strength

Isometric peak force (N) of hip external rotation, abduction, and extension, and trunk, will be measured with a hand held dynamometer (Power Track II Commander Echo; JTECH Medical, Salt Lake City, Utah, USA) as described [30]. During all assessments, a belt will be used to fixate the dynamometer and the participant will be encouraged to push against the dynamometer as much as they can. The leverage (m) will be measured from the joint axis of rotation to the point of application of the force transducer for each test. Each test will be repeated three times and each contraction will be maintained for 5 s with approximately 15 s of rest in between contractions. The peak value of the three trials in Newton meter, normalized for body weight (Nm/kg), will be used in the analysis.

#### 2.3.3 Hop performance

Two reliable hop tests, the single-leg hop for distance and the side-hop, will be performed as described [31]. The participants will perform three to five practice trials followed by three maximum approved trials for the single-leg hop test for distance. The hop distance is measured in centimeters from the toe at the push-off to the heel at the landing position. The side hop will be performed once on each leg and the participants will be instructed to jump as many times as possible, landing outside two tape strips 40 cm apart, during a period of 30 s.

#### 2.3.4 Postural orientation

The test battery consists of 5 functional tasks; single-limb mini squat (SLMS), stair descending (SD), forward lunge (FL), single-leg hop for distance (SLHD) and side hop (SH); tasks ranging from resembling daily to more demanding activities [32]. The tasks will be video-recorded from a frontal view for later assessment of the patient's postural orientation. The videos can be viewed several times and/or in slow motion if needed. Between 2 and 6 segment-specific postural orientation errors (POEs) will be observed and scored for each task on a 4-point ordinal scale from 0 to 3 where 0 = good postural orientation, i.e., represents no signs of POEs, 1 = “fair”, represents signs of POEs, 2 = “poor”, represents clear signs of POEs and 3 = “very poor”, represents when the execution of the test does not have any similarities to the intended task. The total POE score across all and within tasks and segment-specific POEs will be used in the analysis. High inter-rater reliability has been reported for this test battery in patients with ACL injury [32].

#### 2.3.5 Patient-reported outcomes

Data for the following valid questionnaires will be collected and managed using an electronic data capture tool (REDCap) [33] (Lund) or using paper questionnaires (Oslo); The Short-Form 36 [34] will be used for assessment of perceived generic health. For patient-reported knee specific function, the KOOS (all subscales except pain) [35], Global knee function: Numeric Rating Scale (1–10 were 1 = worst and 10 = best) [36]and the ACL Quality of Life [37] will be used. The Tegner activity scale [38] will be used to assess pre-injury and current activity level and the ACL Return to Sport after Injury Scale [39] will be used to evaluate psychological readiness for returning to sport after injury. In addition, the participants will answer a questionnaire regarding their pre-injury activity, if they plan to return/have returned to the same activity or different activity level as well as time point of return (if any). Finally, the Perceived stress scale - 10 [40] will be used for perceived stress.

#### 2.3.6 Muscle activation pattern

For the patients recruited in Lund, muscle activation patterns for the following muscles will be recorded during the landing phase of the single-leg hop for distance, using a wireless electromyographic system (Desktop DTS, Noraxon U.S.A. Inc, Scottdale, Arizona, USA); Gluteus maximus, Gluteus medius, Semitendinosus, Biceps femoris, Vastus medialis, Medial gastrocnemius and Iliocostalis. The maximum voluntary contraction (MVC) for each muscle will be calculated from the maximum value of three repetitions, synchronously collected with the torque data described above.

### 2.4 Outcomes

#### 2.4.1 Patient-reported outcome

The valid, patient-reported KOOS [35] subscale pain, scored on a 0 (worst) to 100 (best) scale will be used as a marker of symptomatic OA. In addition to the continuous measure of KOOS pain, a cut-off of will be used, where a score of ≤72 on the KOOS pain subscale will represent symptomatic knee OA and a score of >72 will represent no symptomatic knee OA, according to that previously described [9,10]. This particular cut-off is based on the normal mean (92.3 ± 10.0) KOOS pain score in an athletic population with a history of knee ligament injury and represents two standard deviations below the mean [41].

#### 2.4.2 MR imaging

Compositional MRI of cartilage quality will be performed using T2 mapping using a 1.5 T scanner.

#### 2.4.3 MR image acquisition

Subjects will be positioned in a supine position with their feet first in a 1.5 T MRI scanner (AvantoFit, Siemens Healthcare, Erlangen, Germany) and the injured knee placed in a knee coil (TxRx 15 Ch Knee, Siemens, Erlangen, Germany). After localizer sequences, four 2D proton density weighted turbo spin echo sequences will be acquired for scoring and clinical evaluation with: 1) sagittal orientation and no fat suppression, and 2–4) sagittal, transversal and coronal orientations with fat suppression. For T2 mapping, two sagittal slices positioned in the central part of each of the medial and lateral tibiofemoral cartilage compartments, respectively, will be acquired in a single, interleaved acquisition using a 2D multi echo spin echo sequence with 12 echoes. Finally, a sagittal T1 weighted 3D VIBE (Volumetric Interpolated Breath Hold Examination) sequence will be acquired for segmentation of the bone. Imaging parameters for each sequence are summarized in Table 3.

**Table 3:** Image acquisition parameters for the proton-density weighted turbo-spin echo (PDW TSE), multi-echo spin echo (MESE) and T1 Weighted Volumetric Interpolated Breath Hold Examination (3D T1W VIBE) sequences.

Sequence	PDW TSE	PDW TSE	PDW TSE	PDW TSE	MESE	T1W VIBE
Orientation	Sagittal	Sagittal	Coronal	Transversal	Sagittal	Sagittal
2D/3D	2D	2D	2D	2D	2D	3D
Fat suppression	None	Fat Sat	Fat Sat	Fat Sat	None	Water excitation
Echo time (ms)	41	45	37	38	10–120	6
Repetition time (ms)^a^	3000	4550	3000	3760	2360	14
Flip angle						10
Turbo factor	10	11	5	5	12	
Field of view (mm^2^)	160×160	170×170	150×150	160×160	145×160	150×150
Voxel size (mm^3^)	0.4×0.4×3	0.5×0.5×3	0.5×0.5×3	0.5×0.5×3	0.4×0.4×3	0.3×0.3×0.6

a May vary slightly as the number of slices will be adjusted for coverage in each case.

#### 2.4.4 Analysis and ROI definition

**Fig. 1:**
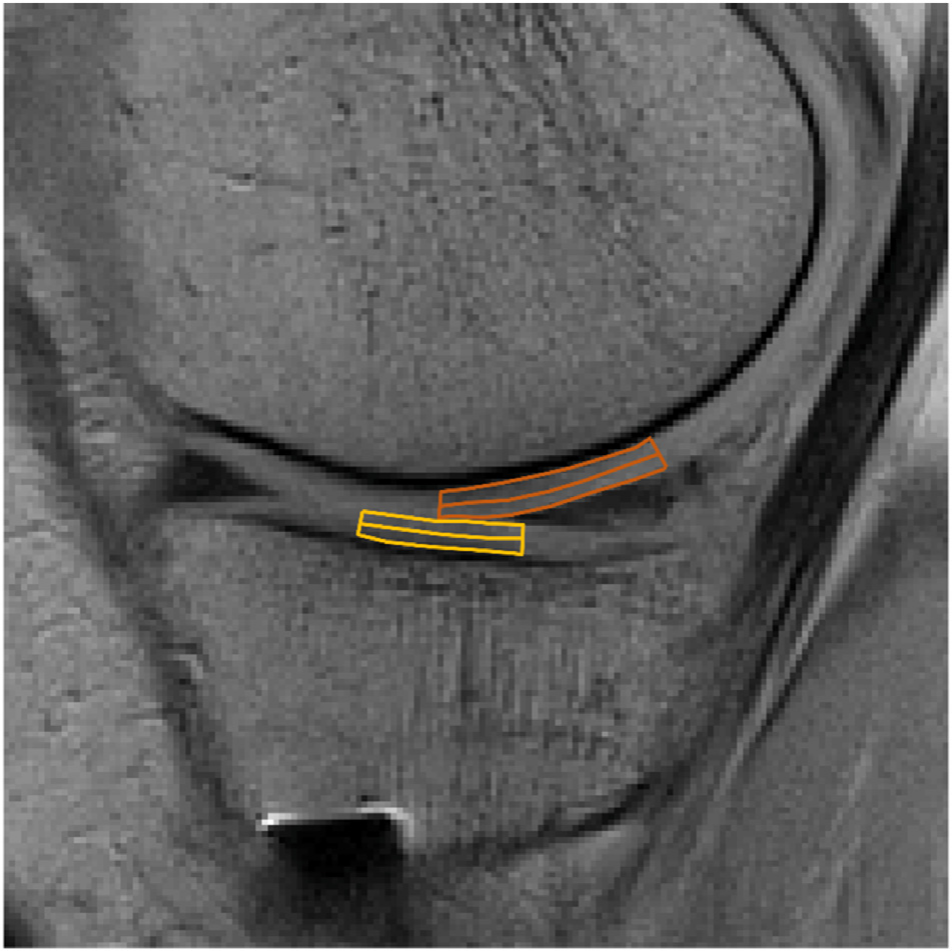
Definition of regions-of-interest (ROIs) in a sagittal view of the medial condyle of an example knee. In both the femoral and tibial cartilages, superficial and deep ROIs will be defined, each covering half of the cartilage thickness. The femoral ROIs (orange) extend from the center of the tibial plateau to the end of the meniscus in the anterior-posterior direction, and the tibial ROIs (yellow) will cover the mid-third of the cartilage width.

T2 maps will be calculated online at the scanner from the acquired multi echo spin echo images using a built-in application (MapIt). Using the ImageJ software (Rasband, W.S., ImageJ, U. S. National Institutes of Health, Bethesda, Maryland, USA, https://imagej.nih.gov/ij/, 1997–2018.), regions of interest (ROIs) will be defined covering the superficial half and the deep half of the cartilage in each of the medial and lateral weight-bearing femoral and tibial cartilages respectively, resulting in a total of eight ROIs per subject MR exam. Although the articular cartilage is generally divided into three layers, distinction beyond a superficial and deep ROI will not be possible here, due to the limited spatial resolution of the MR image. The femoral ROIs will cover a region from the middle of the tibial plateau (regarding anterior-posterior position) to the posterior horn of the meniscus. The tibial ROIs includes the central 1/3 of the tibial cartilage in the sagittal plane (Fig. 1). ROIs will be defined in the shortest TE (TE = 10 ms) image and the position of each ROI will then be copied to the corresponding T2 map for evaluation of T2.

All ROIs will be drawn by a single reader (AC), but the ROIs of a subgroup of 15 subjects will also be drawn by an additional reader (PP), and a second time by AC, for investigation of the inter- and intra-rater reliability, respectively, using the Intra-class Correlation Coefficient (ICC) [42]. Results will be presented as the mean T2 within each ROI, i.e., the deep and superficial regions of the femoral and tibial cartilages, respectively.

#### 2.4.5 Biomarker profiles

Blood samples of venous blood will be collected to assess molecular biomarkers of cartilage and bone turnover in serum [43]. Specific biomarkers including, but not limited to, cartilage markers cartilage oligomeric matrix protein (COMP; ELISA based immunoassay from BioVendor) and ARGS-aggrecan (ARGS neoepitope of aggrecan; in-house immunoassay based on Meso Scale Discovery platform (MSD)), and bone markers N-terminal type I collagen cross-linked telopeptide (NTX-I; ELISA based immunoassay from Osteomark) and tartrate-resistant acid phosphatase 5b (TRAP5b; ELISA based immunoassay from Quidel) will be analysed in batch mode [[44], [45], [46], [47]]. Possibly, inflammatory biomarkers (including C-reactive protein, tumour necrosis factor-α, interleukin-6; e.g. using MSD multiplex immunoassays) will be analysed in batch mode to obtain complementary data [48,49].

### 2.5 Statistical analysis

For descriptive purposes, the mean (SD), or median (quartiles), at baseline and follow-up assessments will be used as appropriate. Separate linear regression models will be used to elucidate the influence of the absolute values of each predictor on the absolute value on primary, secondary and exploratory outcomes in cross-sectional analyses and on the absolute value as well as change in primary, secondary and exploratory outcomes in longitudinal analysis, adjusted for baseline values and possible confounders (e.g., age and associated injuries). In addition, logistic regression analysis will be used to elucidate the influence of each predictor on significant knee pain (KOOS pain ≤72 vs > 72). Assuming a clinically relevant correlation of 0.30 between knee extension strength and self-reported pain [50], we need 84 patients with 80% power at the 5% significance level. Based on this calculation, we will include 100 patients including an approximate drop-out of 15%. For explorative purposes, an analysis of covariance (ANCOVA) will be used to investigate differences in the presence of early knee OA between the 100 patients with ACLR and twenty sex and age matched non-injured individuals, adjusting for activity level.

## 3 Discussion

To our knowledge, our study will be the first to investigate the role of different measures of muscle function as possible protective factors for possible markers of early OA development including patient-reported pain, MRI, and biomarkers in patients with ACLR. There is today no consensus regarding the definition of early symptomatic OA in younger individuals with a history of knee injury [10]. Several different models, such as a cut-off of 72 on the KOOS pain subscale, the minimally clinical reported difference (KOOS pain) and a combination of different cut-offs of the different KOOS subscales have been proposed [10]. In this study, we chose the cut-off of 72 on the KOOS pain subscale since this measure is reported to be related to cartilage loss [11] as well as knee pain in patients with ACL injury [9]. Furthermore, we will include both advanced measures of muscle function (isometric muscle strength assessed with a Biodex) and those that are easily administered in clinical research and in the clinical setting (performance-based measures). If muscle function will be a protective factor for possible markers of early OA, this will have important clinical implications for using muscle function as screening for knee OA at an early stage of the disease, and for optimizing muscle function in the treatment of knee injury. Further studies may determine whether exercise aimed at improving important aspects of muscle function may prevent, or slow the progression of, early signs of knee OA. By addressing muscle function as a modifiable factor to prevent, or slow the progression of, knee OA, this project ultimately has the potential to influence clinical management at an early stage of this chronic disease, and reduce the personal and societal burden of this increasing health problem.

## Authors’ contribution

All authors contributed to the conception and design of this study as well as the writing of this study protocol. All authors read and approved the final version of this manuscript.

## Funding

This study is funded by the Anna-Greta Crafoord Foundation, 10.13039/501100006075Greta and Johan Kock's Foundation The 10.13039/501100007949Swedish Rheumatism Association, The 10.13039/501100006738Faculty of Medicine, The 10.13039/501100005390Alfred Österlund Foundation, The 10.13039/501100003173Crafoord Foundation, The 10.13039/501100006285Magnus Bergvall Foundation and Governmental Funding of Clinical Research within the National Health Service (ALF).

## Declaration of competing interest

The authors declare no competing interest.

## Figures and Tables

**Table A1:** ICC values of the muscle strength measures and hop tests.

Test	ICC_2*K*_
Hip external rotation peak torque (hhd)	0.931
Hip extension peak torque (hhd)	0.666
Hip abduction peak torque (hhd)	0.759
Side-bridge peak torque (hhd)	0.726
Knee extension peak torque (biodex)	0.849
Knee flexion peak torque (biodex)	0.838
SLHD (cm)	1.0
Side-hop (n)	0.994

ICC = intra-class correlation coefficient, hhd = hand-held dynamometer.

SLHD = single-leg hop for distance, cm = centimeter, n = number of repetitions.

## Appendix A Assessment of inter-rater reliability

### Methods

An inter-rater reliability assessment between the two testers in Sweden and Norway was performed in November 2017. Ten healthy individuals without previous knee injury performed all muscle strength tests, single-leg hop for distance and the side-hop as described in the study protocol. A 5-min warm-up on a stationary bike was performed prior to the tests. The tests were performed twice with 30 min of rest between tests. Two minimize bias relating to learning effects, half of the participants were assessed by one of the testers on their first testing session and the other half were assessed by the second tester on their first testing session.

### Results

A moderate to excellent inter-rater reliability were found for all assessments (ICC_2*K*_ 0.66–1.0) (Table A1).
